# Supplementary figures and images for: Impact of a perioperative oral opioid substitution protocol during the nationwide intravenous opioid shortage: A single center, interrupted time series with segmented regression analysis
Source: PLoS One. 2020 Jun 4;15(6):e0234199. doi: 10.1371/journal.pone.0234199 (PMC7272091; doi:10.1371/journal.pone.0234199)

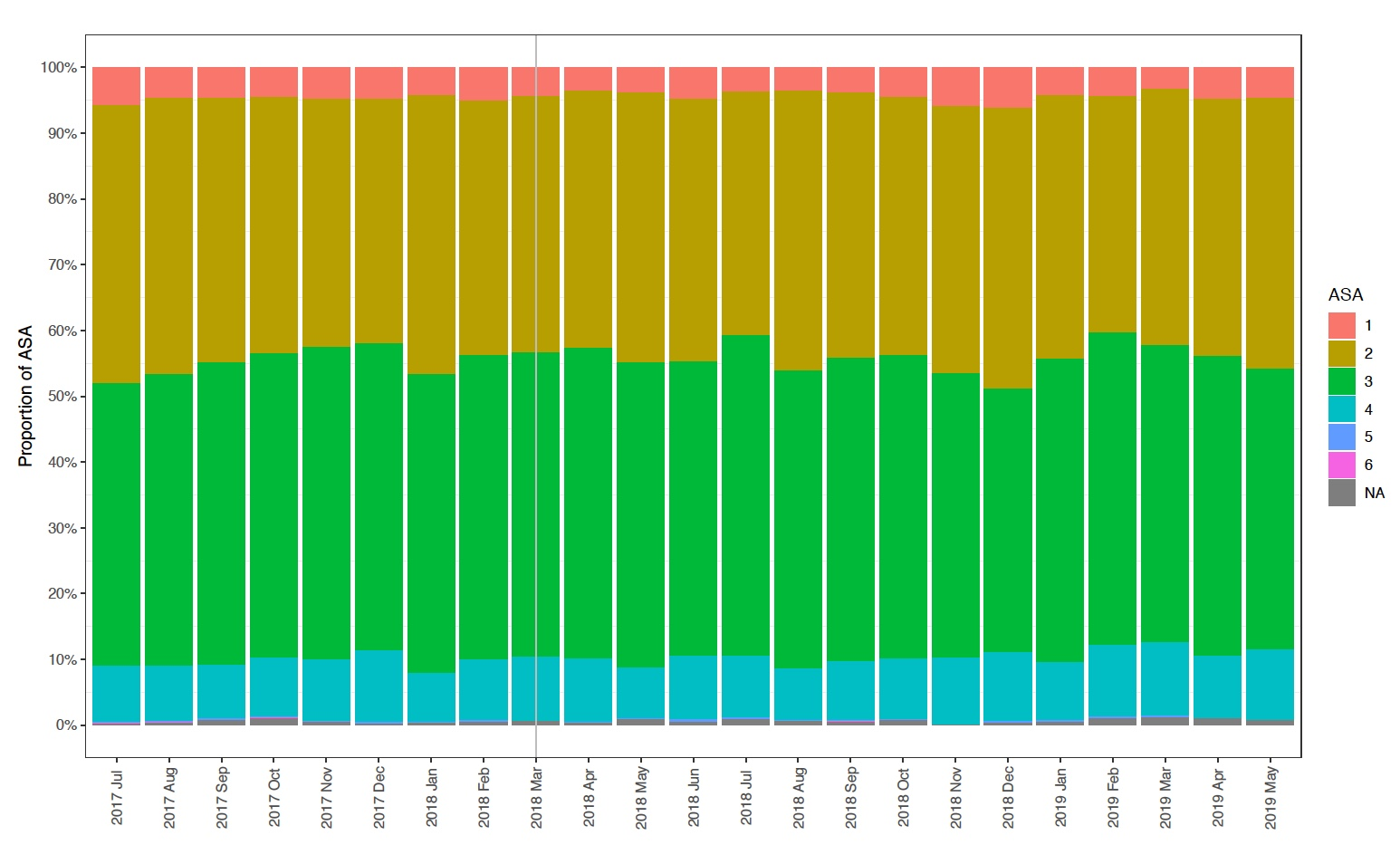

Supplement: S1 Fig — NA: Cases with missing ASA classification. (TIFF) [file pone.0234199.s002.tiff]

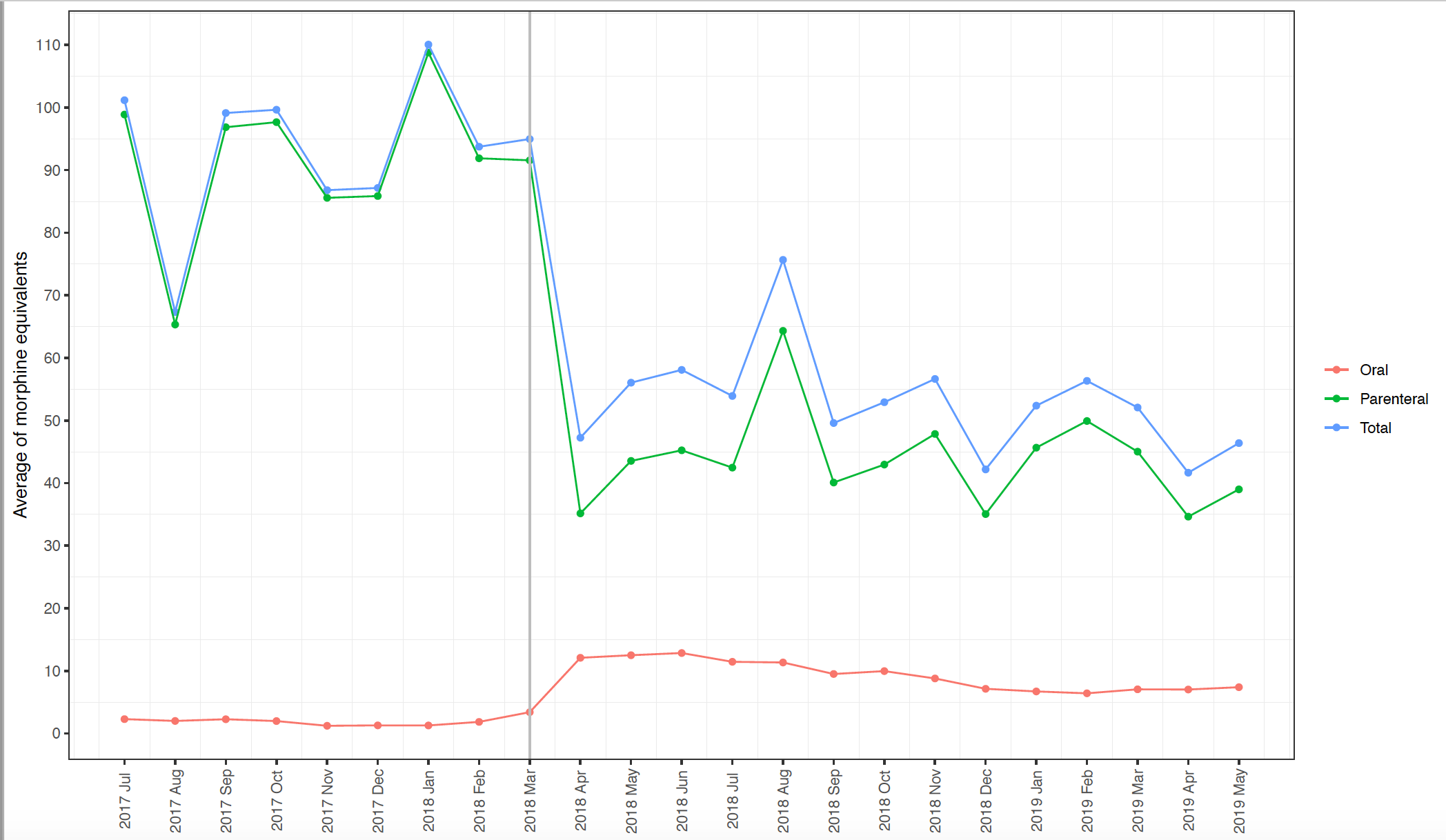

Supplement: S2 Fig — (TIFF) [file pone.0234199.s003.tiff]

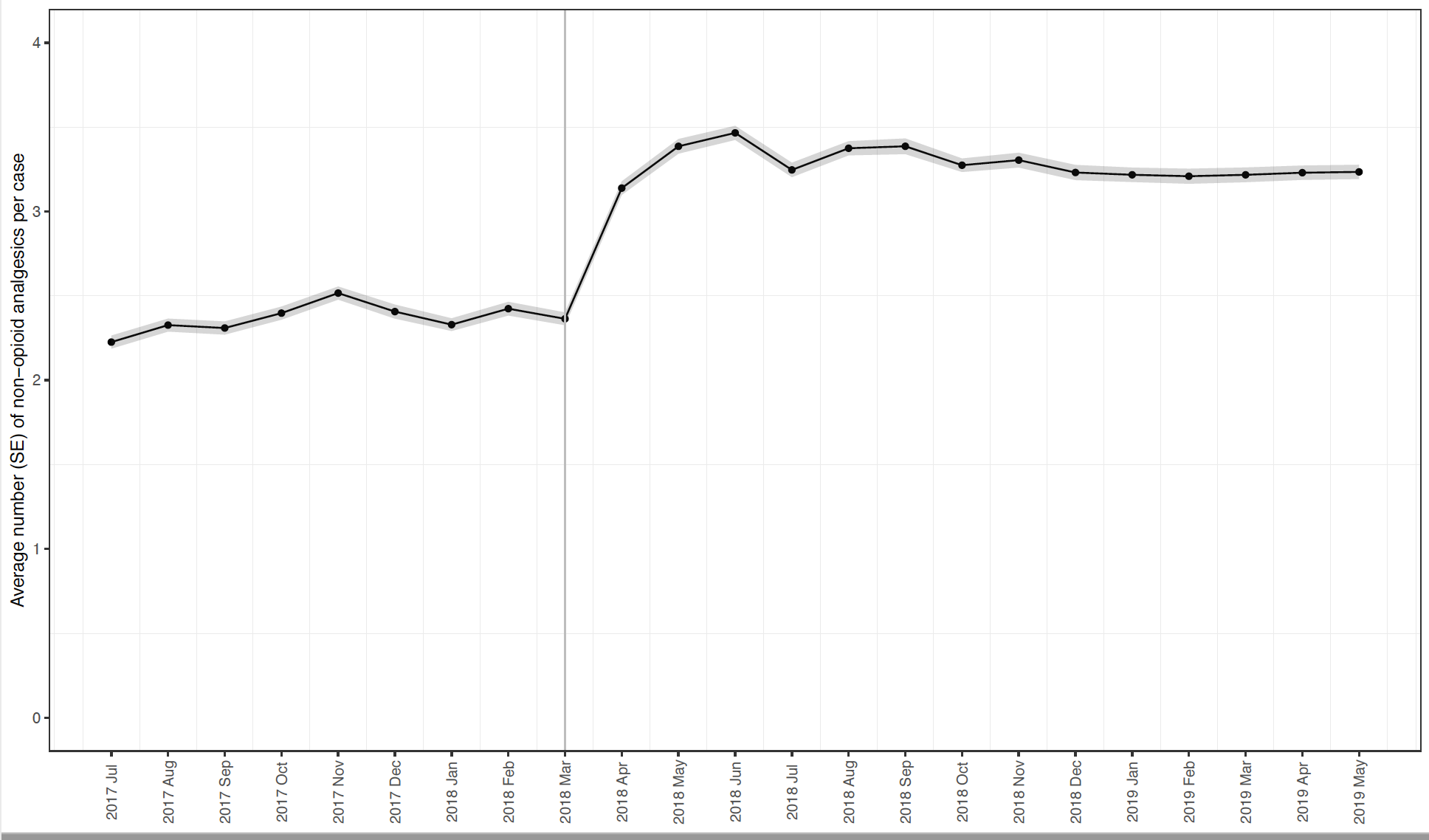

Supplement: S3 Fig — (TIFF) [file pone.0234199.s004.tiff]

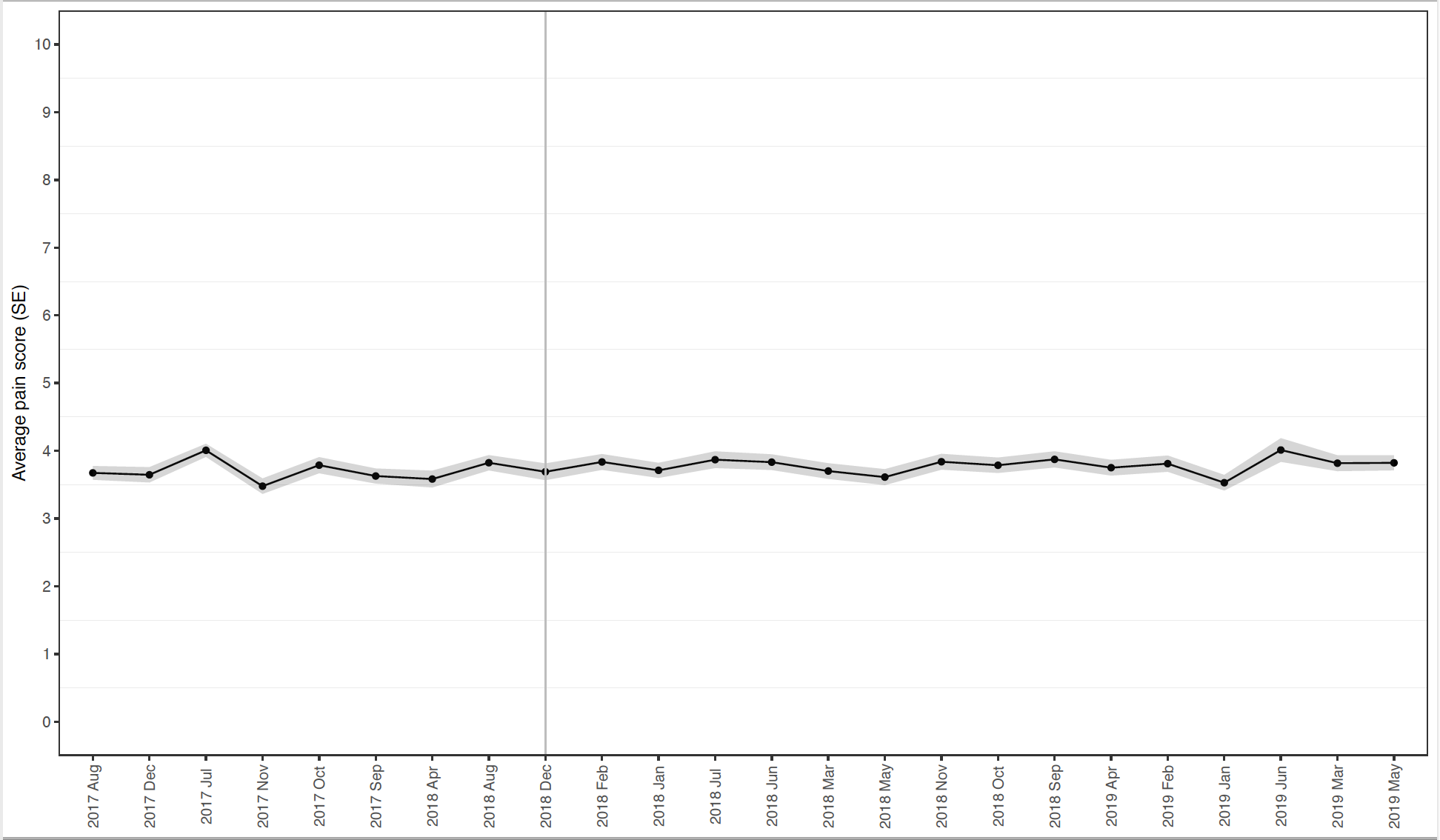

Supplement: S4 Fig — (TIFF) [file pone.0234199.s005.tiff]

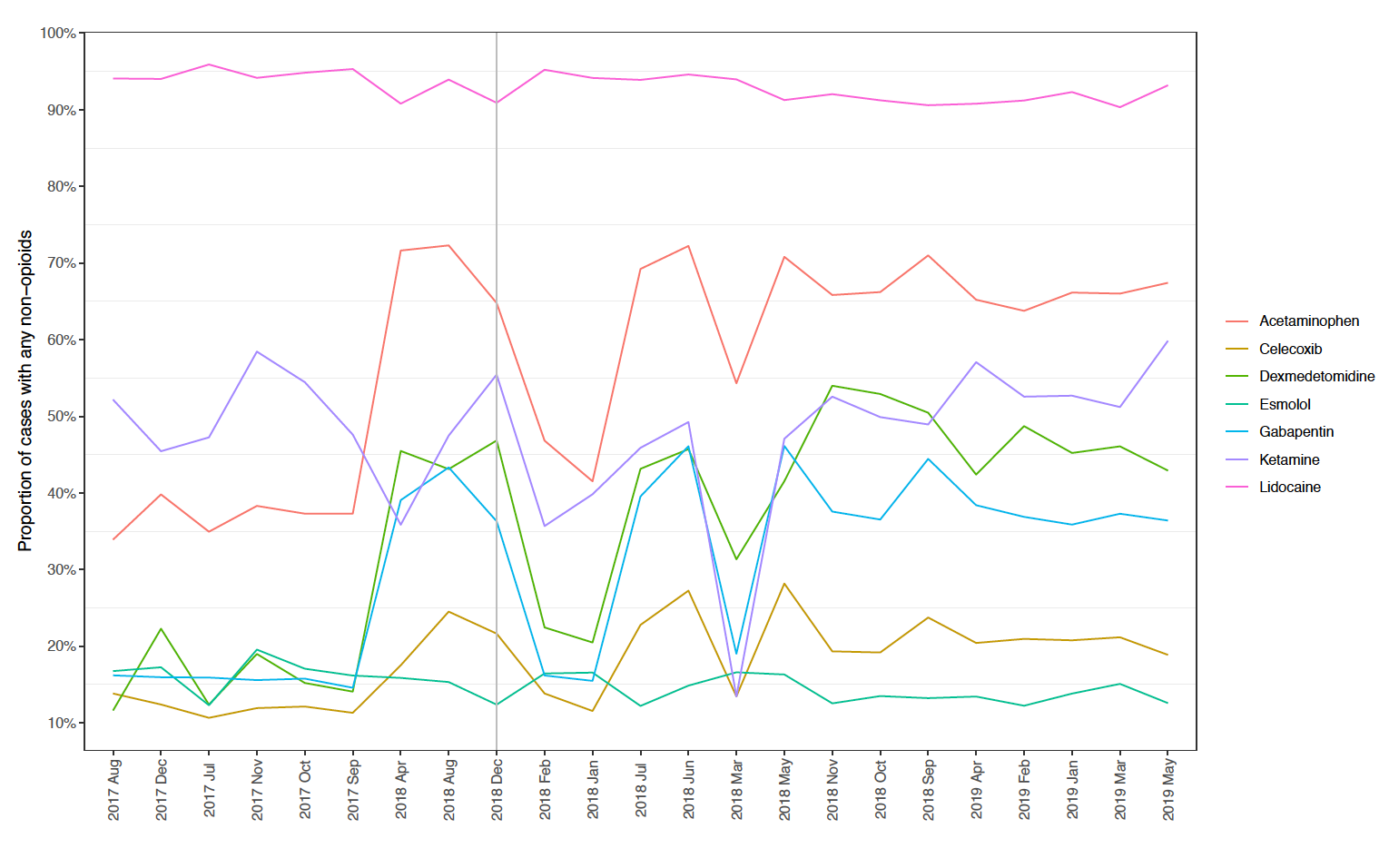

Supplement: S5 Fig — (TIFF) [file pone.0234199.s006.tiff]

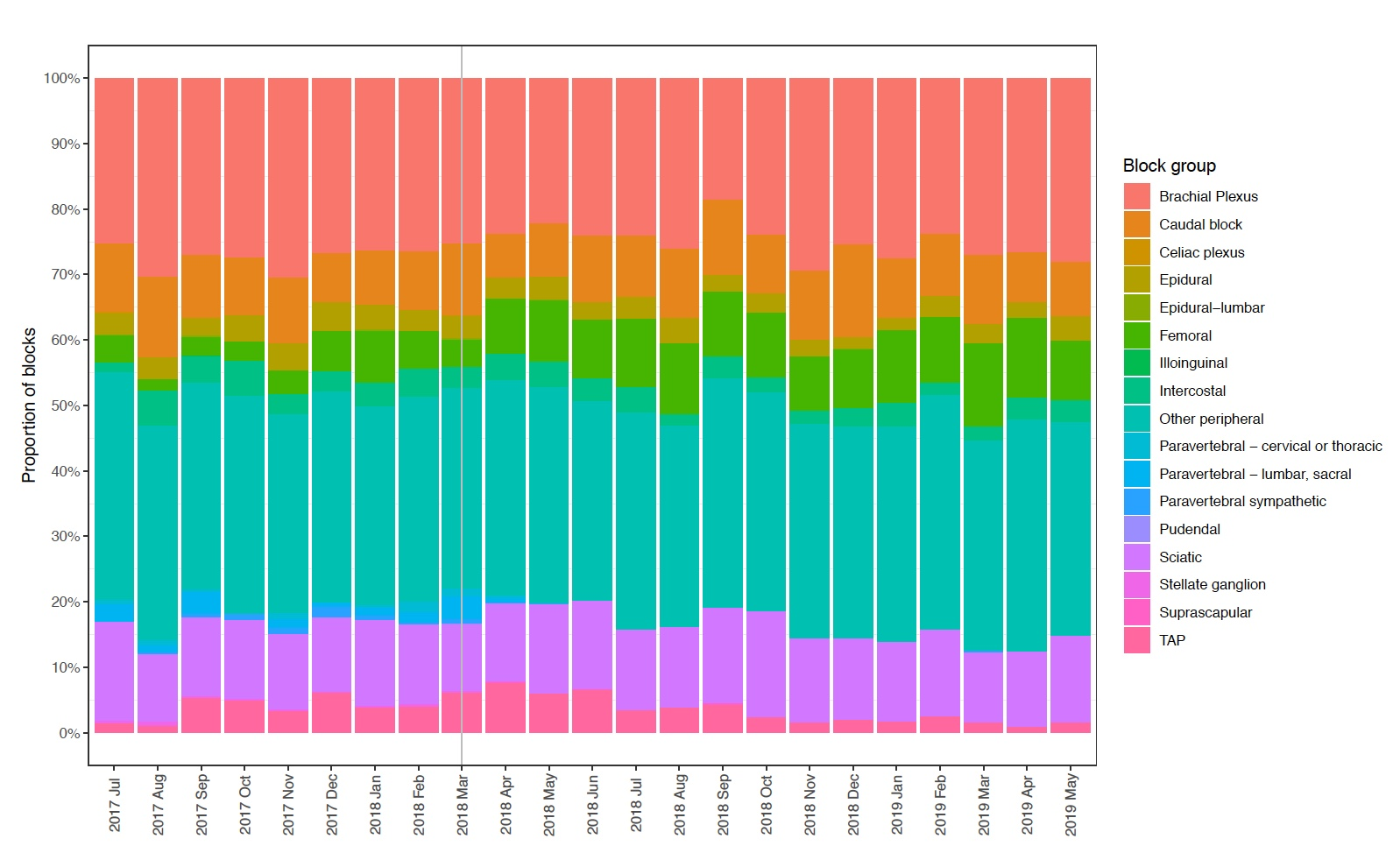

Supplement: S6 Fig — (TIFF) [file pone.0234199.s007.tiff]
