# Supplementary material for: Impact of a perioperative oral opioid substitution protocol during the nationwide intravenous opioid shortage: A single center, interrupted time series with segmented regression analysis
Source: PLoS One. 2020 Jun 4;15(6):e0234199. doi: 10.1371/journal.pone.0234199 (PMC7272091; doi:10.1371/journal.pone.0234199)
Supplement: S1 Table — Pre-pre intervention, post-post intervention. (DOCX) [file pone.0234199.s008.docx]

|  |  | **Total Morphine Equivalents (mg)** | | | **Oral Morphine Equivalents (mg)** | | | **Parenteral Morphine Equivalents (mg)** | | |
| --- | --- | --- | --- | --- | --- | --- | --- | --- | --- | --- |
| **Month/Year** | **Pre/Post** | **Mean (SD)** | **Median [IQR]** | **Min-Max** | **Mean (SD)** | **Median [IQR]** | **Min-Max** | **Mean (SD)** | **Median [IQR]** | **Min-Max** |
| July, 2017 | Pre | 101 (632) | 30 [51] | 0-17,869 | 2 (6) | 0 [0] | 0-67 | 98(632) | 30 [49] | 0-17,869 |
| August, 2017 | Pre | 67 (218) | 30 [45] | 0-4,449 | 2 (5) | 0 [0] | 0-30 | 65(218) | 30 [43] | 0-4,449 |
| September, 2017 | Pre | 99 (511) | 30 [51] | 0-14,418 | 2 (16) | 0 [0] | 0-540 | 96(511) | 30 [49] | 0-14,418 |
| October, 2017 | Pre | 99 (409) | 33 [51] | 0-7,401 | 2 (5) | 0 [0] | 0-45 | 97 (409) | 30 [48] | 0-7,401 |
| November, 2017 | Pre | 86 (296) | 30 [49] | 0-5,067 | 1 (4) | 0 [0] | 0-30 | 85(296) | 30 [48] | 0-5,067 |
| December, 2017 | Pre | 87 (321) | 33 [51] | 0-6,920 | 1 (4) | 0 [0] | 0-40 | 85(322) | 30 [48] | 0-6,920 |
| January, 2018 | Pre | 110 (437) | 30 [48] | 0-6, 814 | 1 (4) | 0 [0] | 0-45 | 108(438) | 30 [45] | 0-6, 806 |
| February, 2018 | Pre | 93 (430) | 31 [48] | 0-11, 596 | 1 (4) | 0 [0] | 0-45 | 91(430) | 30 [49] | 0-11, 596 |
| March, 2018 | Pre | 94 (439) | 30 [52] | 0-9, 052 | 3 (14) | 0 [0] | 0-420 | 91(439) | 30 [57] | 0-9, 052 |
| April, 2018 | Post | 47 (290) | 15 [30] | 0-7,530 | 12 (16) | 7.5[15] | 0-90 | 35(290) | 0 [15] | 0-7,500 |
| May, 2018 | Post | 56 (255) | 15 [37] | 0-4,041 | 12 (18) | 7.5[15] | 0-135 | 43(254) | 0 [22] | 0-4,026 |
| June, 2018 | Post | 58 (347) | 15 [44] | 0-6,567 | 12 (18) | 7.5[15] | 0-120 | 45(347) | 0 [30] | 0-6,544 |
| July, 2018 | Post | 53 (219) | 15 [45] | 0-4,508 | 11 (16) | 7.5[15] | 0-105 | 42(218) | 0 [30] | 0-4,508 |
| August, 2018 | Post | 75 (401) | 15 [44] | 0-7,294 | 11 (17) | 7.5[15] | 0-270 | 64(402) | 0.75 [30] | 0-7,294 |
| September, 2018 | Post | 49 (205) | 15 [37] | 0-3,123 | 10 (15) | 0 [15] | 0-135 | 40(204) | 0.75 [30] | 0-3,108 |
| October, 2018 | Post | 52 (192) | 15 [45] | 0-3,607 | 10 (30) | 0 [15] | 0-1056 | 42(189) | 7.5 [30] | 0-3,607 |
| November, 2018 | Post | 56 (324) | 15 [36] | 0-9,263 | 9 (18) | 0 [15] | 0-450 | 47(324) | 7.5 [30] | 0-9,263 |
| December, 2018 | Post | 42 (182) | 15 [34] | 0-4,535 | 7 (11) | 0 [15] | 0-90 | 35(181) | 7.5 [30] | 0-4,535 |
| January, 2019 | Post | 52 (209) | 15 [35] | 0-2,859 | 7 (11) | 0 [15] | 0-120 | 45(209) | 6.5 [30] | 0-2,844 |
| February, 2019 | Post | 56 (303) | 15 [30] | 0-5,543 | 6 (11) | 0 [15] | 0-90 | 49(304) | 7.5[ 24] | 0-5,543 |
| March, 2019 | Post | 52 (277) | 15 [30] | 0-5,304 | 7 (11) | 0 [15] | 0-165 | 45(277) | 6 [22] | 0-5,274 |
| April, 2019 | Post | 41 (142) | 15 [34] | 0-2,290 | 7 (11) | 0 [15] | 0-105 | 34(142) | 7.5 [22] | 0-2,290 |
| May, 2019 | Post | 46 (192) | 15 [36] | 0-3,770 | 7 (11) | 0 [15] | 0-90 | 39(192) | 7.5 [24] | 0-3,770 |

Supplementary Table 1: Mean (standard deviation), median[interquartile range] and minimum and maximum total, oral and parenteral morphine equivalents in the pre and post intervention period. Pre-pre intervention, post-post intervention.
