# Supplementary material for: Impact of a perioperative oral opioid substitution protocol during the nationwide intravenous opioid shortage: A single center, interrupted time series with segmented regression analysis
Source: PLoS One. 2020 Jun 4;15(6):e0234199. doi: 10.1371/journal.pone.0234199 (PMC7272091; doi:10.1371/journal.pone.0234199)
Supplement: S2 Table — Data presented as percentage and percentage change from previous month. Pre-pre intervention, post-post intervention. (DOCX) [file pone.0234199.s009.docx]

| **Month/Year** | **Pre/Post** | **Percent (Percent change from previous month)** | | | | | | |
| --- | --- | --- | --- | --- | --- | --- | --- | --- |
|  | | **Acetaminophen** | **Celecoxib** | **Dexmedetomidine** | **Esmolol** | **Gabapentin** | **Ketamine** | **Lidocaine** |
| July, 2017 | Pre | 35 | 11 | 12 | 12 | 16 | 47 | 96 |
| August, 2017 | Pre | 34 (-3) | 14 (30) | 12 (-6) | 17 (36) | 16 (2) | 52 (10) | 94 (-2) |
| September, 2017 | Pre | 37 (10) | 11(-18) | 14 (21) | 16(-3) | 15 (-10) | 48 (-9) | 95 (1) |
| October, 2017 | Pre | 37 (0) | 12(7) | 15 (8) | 17(6) | 16 (8) | 54(14) | 95 (-1) |
| November, 2017 | Pre | 38 (3) | 12(-2) | 19 (25) | 20 (15) | 16 (-1) | 58 (7) | 94 (-1) |
| December, 2017 | Pre | 40 (4) | 12(4) | 22 (17) | 17 (-12) | 16 (2) | 45 (-22) | 94 (0) |
| January, 2018 | Pre | 42 (4) | 12(-7) | 20 (-8) | 17 (-4) | 15 (-3) | 40 (-12) | 94 (0) |
| February, 2018 | Pre | 47 (13) | 14 (20) | 22 (10) | 16 (-1) | 16 (5) | 36 (-10) | 95 (1) |
| March, 2018 | Pre | 54 (16) | 13 (-3) | 31 (40) | 17 (1) | 19 (17) | 13 (-62) | 94 (-1) |
| April, 2018 | Post | 72 (32) | 18 (30) | 45 (45) | 16 (-4) | 39 (105) | 36 (166) | 91 (-3) |
| May, 2018 | Post | 71(-1) | 28 (61) | 42 (-9) | 16 (3) | 46 (18) | 47 (31) | 91 (1) |
| June, 2018 | Post | 72(2) | 27 (-3) | 46 (10) | 15 (-9) | 46 (0) | 49 (5) | 95 (4) |
| July, 2018 | Post | 69(-4) | 23 (-16) | 43 (-6) | 12 (-18) | 40 (-14) | 46 (-7) | 94 (-1) |
| August, 2018 | Post | 72(4) | 25 (8) | 43 (0) | 15 (26) | 43 (10) | 47 (3) | 94 (0) |
| September, 2018 | Post | 71(-2) | 24 (-3) | 50 (17) | 13 (-14) | 44 (3) | 49 (3) | 91 (-4) |
| October, 2018 | Post | 66 (-7) | 19 (-19) | 53 (5) | 14 (2) | 37 (-18) | 50 (2) | 91 (1) |
| November, 2018 | Post | 66 (-1) | 19 (1) | 54 (2) | 13 (-7) | 38 (3) | 53 (5) | 92 (1) |
| December, 2018 | Post | 65 (-2) | 22 (12) | 47 (-13) | 12 (-1) | 36 (-3) | 55 (5) | 91 (-1) |
| January, 2019 | Post | 66 (2) | 21 (-4) | 45 (-3) | 14 (12) | 36 (-1) | 53 (-5) | 92 (2) |
| February, 2019 | Post | 64 (-4) | 21 (1) | 49 (8) | 12 (-11) | 37 (3) | 53 (0) | 91 (-1) |
| March, 2019 | Post | 66 (4) | 21 (1) | 46 (-5) | 15 (23) | 37 (1) | 51 (-3) | 90 (-1) |
| April, 2019 | Post | 65 (-1) | 20 (-4) | 42 (-8) | 13 (-11) | 38 (3) | 57 (11) | 91 (1) |
| May, 2019 | Post | 67 (3) | 19 (-8) | 43 (1) | 13 (-6) | 36 (-5) | 60 (5) | 93 (3) |

Supplementary Table 2: Changes in the percentage of nonopioids administered pre and post intervention. Data presented as percentage and percentage change from previous month. Pre-pre intervention, post-post intervention.
