# Supplementary material for: Impact of a perioperative oral opioid substitution protocol during the nationwide intravenous opioid shortage: A single center, interrupted time series with segmented regression analysis
Source: PLoS One. 2020 Jun 4;15(6):e0234199. doi: 10.1371/journal.pone.0234199 (PMC7272091; doi:10.1371/journal.pone.0234199)
Supplement: S3 Table — TAP: Transverse abdominis plane. (DOCX) [file pone.0234199.s010.docx]

| **Block Type** | **Pre-Intervention** | **Post-Intervention** | ***p*** |
| --- | --- | --- | --- |
| Brachial Plexus | 27.2 + 1.9 | 24.9 + 2.7 | 0.04 |
| Caudal block | 9.7+ 1.5 | 9.7 + 1.9 | 0.98 |
| Celiac plexus | 0.04 + 0.1 | 0 + 0 |  |
| Epidural | 3.5 + 0.5 | 2.9 + 0.6 | 0.02 |
| Epidural-lumbar | 0.1 + 0.1 | 0.02 + 0.1 |  |
| Femoral | 4.4 + 1.9 | 10.0 + 1.3 | < 0.001 |
| Illo-inguinal | 0.02 + 0.1 | 0 + 0 |  |
| Intercostal | 3.7 + 1.2 | 2.9 + 0.8 | 0.09 |
| Other peripheral | 31.9 + 1.5 | 33.1 + 1.6 | 0.09 |
| Paravertebral - cervical or thoracic | 0.7 + 0.5 | 0.03 + 0.1 |  |
| Paravertebral - lumbar, sacral | 1.7 + 1.2 | 0.04 + 0.2 |  |
| Paravertebral sympathetic | 0.7 + 0.5 | 0.03 + 0.1 |  |
| Pudendal | 0.02 + 0.1 | 0 + 0 |  |
| Sciatic | 11.9 + 1.5 | 12.9 + 1.4 | 0.13 |
| Stellate ganglion | 0.3 + 0.1 | 0.01 + 0.04 |  |
| Suprascapular | 0 + 0 | 0.04 + 0.1 |  |
| TAP | 4.1 + 1.8 | 3.3 + 2.1 | 0.39 |

Supplementary Table 1: Changes in the proportion of block groups pre and post intervention. TAP: Transverse abdominis plane
