# Supplementary material for: Impact of a perioperative oral opioid substitution protocol during the nationwide intravenous opioid shortage: A single center, interrupted time series with segmented regression analysis
Source: PLoS One. 2020 Jun 4;15(6):e0234199. doi: 10.1371/journal.pone.0234199 (PMC7272091; doi:10.1371/journal.pone.0234199)
Supplement: Attachment — Submitted filename: Response to reviewers .docx [file pone.0234199.s013.docx]

**Response to reviewers**

Reviewer #1: The authors report an interesting large cohort study. Due to an unanticipated shortage of intravenous opioids, the authors had to follow the multimodal non-opioid analgesia procedures widely described in the literature. Their results are in line with all the literature. They confirm the potential hyperalgesia induced by opioids.
If the segmented regression analysis is interesting, it remains difficult to follow to be didactic. This aspect needs to be improved.
+ Give results not only with mean (SE) but also with SD and with median (IQR), Max and

In the revised manuscript, we included median, minimum, and maximum values in the descriptive statistics (Table 1). We have also provided an additional table (Supplementary Table 1) that reports the mean (standard deviation), median[interquartile range] and minimum-maximum for total, parenteral and oral opioids administered as requested by the reviewer. Results of the interrupted time series analyses are presented as means and standard errors (SEs). The SEs reflect the precision of the estimates (i.e., the means). We disagree with this reviewer’s suggestion to include standard deviations (SDs), as SDs reflect only the variability of a variable, not the precision of the estimated mean.

+ Has dexamethasone been used in some cases?

Dexamethasone was not included in this analysis. However, we are aware of several studies that have documented the small but statistically significant reduction in opioid use with dexamethasone. We have included this as a limitation in the ‘Discussion’.

+ Fig 1: summary of ASA classification as not significant; but put the proportions of the different surgeries (not as supplemented data).

We have added the proportions of different surgical services in the manuscript (**Fig 2: Proportion of surgical procedures over time)**

Is it possible to have the level of preoperative pain, if there is opioid tolerant patient and so (beta-blockers …)?

Thank you for this insightful comment. Preoperative pain scores were not reliably collected before surgery therefore we did not include them in the analysis. We have added this to the limitations in the ‘Discussion’ (Page 21, line 354-357).

Differentiate the presentation in tables with a "classic" presentation and the figs in trend curves.

In the revised manuscript, we included descriptive statistics by month and additional figures in the supplementary material to illustrate the trends in the data (Supplementary Fig 2-5).

+ What is the possible explanation for a reduced length of time (duration of surgery Vs. duration of anesthesia timing?).

It’s important to differentiate a ‘statistically’ significant result in such a large dataset vs. the clinically meaningful effect size. The effect size of case length here is 4.5 (1.33) minutes, which is not clinically meaningful in our opinion. This is elaborated in the first paragraph of the Results section.

A correlation graph between the amount of morphine used during the anesthesia and postoperatively would be useful.

We did not parse out the cumulative morphine requirements by pre/intraoperative and post anesthesia care unit. This is primarily due to difficulty in anchoring dose administration for these different time periods due to how they are recorded in the electronic medical record. We therefore did not perform this analysis however we recognize the importance of this analysis.

A presentation of the potential correlation between postoperative pain and postoperative consumption of morphine would also be useful (in PACU and on the floor).

We did not collect any medication data or pain scores from the wards.

In the same line, can the side effects related to opioids (such as PONV or at least their treatment) and to the molecules used in substitution (such as bradycardia, sedation) be reported? Was there an effect on the length of hospital stay for the same surgical procedure?

We did not collect data for complications in this study. Furthermore, the study was confined to the intraoperative and immediate postoperative period only. The primary goal of this study was to present a framework for the use of oral opioids supplemented by nonopioid analgesics in the event of reduced intravenous opioid availability (example during the current COVID pandemic)

+ Because the intraoperative paradigm shift (opioid-based anesthesia to opioid-reduced anesthesia or opioid-free anesthesia) which reactivity (i.e. pain) monitoring was used during the anesthesia?

The indication for administering intraoperative intravenous opioids are described in the section: Intraoperative Phase of Care (Point 8) on page 9, ln 156-157 and ln 161-162. Primarily hypertension and tachycardia, after excluding inadequate depth of anesthesia, was the trigger to administer intravenous opioids.

Reviewer #2: In my opinion, an analysis of the non-opioid agents that are used to make morphine sparing is missing.

Thank you for this excellent suggestion. We have provided both a table (Supplementary Table 2) and trend graph of nonopioid analgesics (Supplementary Figure 5) administered during the pre and post-intervention. This provides the reader with information on how the frequency of nonopioid analgesic use changed during the pre and post intervention period.

Indeed, the message in the present state is that morphine sparing is mainly a cultural problem and that by using more non-morphine agents the immediate perioperative consumption of morphine is reduced.

We humbly disagree with the reviewer on this point. We feel that there are two factors at play: increased nonopioid use (on average one additional nonopioid use) and the phenomena of reduced opioid induced hyperalgesia with less total intraoperative opioid used, as highlighted by reviewer 1.

There is not enough development on the molecules that need to be used more (dexmedetomidine according to your data?). The dose of the drug is not raised, but it is likely that some (such as ketamine) will be used at higher doses, which I think should be said in the discussion.

We have provided an ITS on the number of nonopioid analgesics administered during the case. Per the reviewer’s earlier suggestion, we have provided both a table and trend graph of nonopioid analgesics administered during the pre and post-intervention. This provides the reader with data on how the frequency of nonopioid analgesic use changed during the pre and post intervention period. Doses of medications were not changed during the post intervention period.
