# Supplementary material for: Impact of a perioperative oral opioid substitution protocol during the nationwide intravenous opioid shortage: A single center, interrupted time series with segmented regression analysis
Source: PLoS One. 2020 Jun 4;15(6):e0234199. doi: 10.1371/journal.pone.0234199 (PMC7272091; doi:10.1371/journal.pone.0234199)
Supplement: Attachment — Submitted filename: Rresponse to reviewers-R2.docx [file pone.0234199.s014.docx]

Thank you to the reviewer for the comments regarding our first revision. We have provided a point by point response to his comments.

Reviewer #1:

I carefully reread the revised version of the manuscript, the questions and the answers. I am perplexed about the didactic interest of this work. Indeed, if the evolution was made due to a restriction of the parenteral opioid stock, it also corresponds to the classic evolution of practices with a more extensive use of non-opioid analgesic drugs. This current work confirms these results: more non-opioid used allows less opioid for, at least, the same effectiveness.

Response: We agree with the reviewer that increased nonopioid analgesic use (based on a protocol) definitely has an opioid sparing effect. This study confirms this well described effect. However, the study also elucidates the comparative benefits of an oral opioid regimen that can be utilized when parenteral opioids are limited. Furthermore, our study also tentatively explores the effect of opioid induced hyperalgesia caused by high dose parenteral opioids, which may be mitigated by oral opioids. This is reported extensively in the ‘Discussion’ section.

If we look carefully at the modifications with the non-opioid molecules; it seems to have been introduced before the change in practice (see: dexmedetomidine, acetaminophen and celecoxib) or without any change (lidocaine, esmolol).

Response: The changes in nonopoid use varied slightly between agents but Supplementary table 2 clears demonstrates the % changes when the protocol was formally started in April, 2018 compared to the previous month[Acetaminophen (54 to 72%), celecoxib (13 to 18-28%), dexmedetomidine (31 to 45%).

Would this be due only to the change in the route of administration of opioids (i.e. oral Vs. parenteral)? I’m not so sure.

Response: Our study tentatively explores the effect of opioid induced hyperalgesia caused by high dose parenteral opioids, which may be mitigated by oral opioids. We discuss the findings of the study by Ackerman et al who demonstrated reduced opioid use when they transitioned from a parenteral to oral opioid regimen (Page 20, line 331-345). This is reported extensively in the ‘Discussion’ section.

Minor points.

+ The use of gabapentin preoperatively is less and less recommended on the basis of meta-analysis.

Response: We agree with the reviewer and have included this in our Discussion (Page 20, line 318-321)

+ P7 L1119-120 “prior to surgery, add during the procedure, and in the PACU”

Response: We have added this to the manuscript. Page 7, line 120-121

+ Can't the fact that the patients were significantly older, with significantly shorter procedures, be responsible for these results?

Response: We humbly disagree with the reviewer for the following reasons: 1) the cohort size for this study is large (n=29, 621), therefore small changes may be statistically significant. However, it is important to interpret the effect size of the change in age and procedure length, which is included in Table 1. The effect size for age is 0.61 years, while the procedure length is 4.49 minutes. These changes are not clinically relevant. The following reference is critical to understanding our response : Using Effect Size—or Why the P Value Is Not Enough.

J Grad Med Educ. 2012 Sep; 4(3): 279–282. PMID: 23997866. We have addressed this on page 12, line 217-221.
